# Association of sickle cell trait with β‐cell dysfunction and physical activity in adults living with and without HIV in Tanzania

**DOI:** 10.1111/apm.13214

**Published:** 2022-03-01

**Authors:** Belinda V. Kweka, Cyprian Fredrick, Brenda Kitilya, Kidola Jeremiah, Eric Lyimo, Suzanne Filteau, Andrea M. Rehman, Henrik Friis, Mette F. Olsen, Daniel Faurholt‐Jepsen, Rikke Krogh‐Madsen, George PrayGod

**Affiliations:** ^1^ Mwanza Research Centre National Institute for Medical Research Mwanza Tanzania; ^2^ Faculty of Epidemiology and Population Health London School of Hygiene and Tropical Medicine London UK; ^3^ Department of Nutrition, Exercise, and Sports University of Copenhagen Copenhagen Denmark; ^4^ Department of Infectious Diseases Rigshospitalet Copenhagen Denmark; ^5^ Department of Infectious Diseases Copenhagen University Hospital Hvidovre, Copenhagen Denmark

**Keywords:** Sickle cell trait, HIV, antiretroviral therapy, β‐cell dysfunction and physical activity

## Abstract

This study aimed to investigate sickle cell trait (SCT) associations with physical activity, markers of insulin secretion and resistance, and glucose among people living with HIV infection (PLWH), both antiretroviral therapy (ART) naive and experienced, and HIV‐uninfected adults. This was a cross‐sectional study conducted in Mwanza, Northwestern Tanzania. We used data of 668 participants attained from two sub‐studies of CICADA study. Mean age was 40 (SD 11.5) years, 402 (61.7%) were females and 157 (24.1%) had SCT. PLWH were 422 (64.7%), of these, 80 (18.9%) were on ART. People with SCT had higher risk of having an isolated β‐cell dysfunction compared to those without SCT (RRR = 1.82, CI: 1.10, 3.01, p = 0.02). People with SCT but without HIV infection had lower average acceleration on the trunk longitudinal axis (ACCx) and higher level of self‐reported physical activity. 30 min oral glucose tolerance test among PLWH on ART was higher in those with SCT compared to those without SCT. People with SCT are at higher risk of having β‐cell dysfunction and those with SCT on ART are at more risk of developing diabetes. Future studies to investigate the interaction between SCT and HIV/ART on risk of diabetes should be considered.

Sickle cell trait (SCT) is the heterozygous form of sickle cell disease (SCD) most prevalent in Sub‐Saharan Africa (SSA) where it affects about a quarter of the population [1, 2, 3]. SCD is one of the most common and severe inherited red blood cell disorder caused by a structural change of normal hemoglobin (HbA) to abnormal hemoglobin called sickle hemoglobin (HbS) accompanied with a number of complications, including severe anemia, renal impairment, stroke, and pain [4, 5]. HbSS, the severe type of SCD, occurs when a person inherits two sickle cell genes (“S”), one from each parent [6], while HbAS, the heterozygous form, is the common type of SCT [7].

Unlike SCD, SCT is regarded as less important because people with this disorder present with no or minimal symptoms, are expected to live normal lives, and have the same life span as people without the trait [8]. Despite this, SCT has been reported to be associated with higher risk of cardiovascular diseases, ischemic stroke, deep vein thrombosis, and chronic kidney disease [9, 10, 11]. In addition, complications like hemolysis and autoxidation are estimated to be two times higher in people with HbS traits (e.g., HbSS, HbAS, etc) compared to those with HbAA [12, 13, 14, 15]. An increased rate of hemolysis and autoxidation of HbS causes overproduction of reactive oxygen species (ROS) leading to oxidative stress [15, 16]. High level of ROS may overwhelm protective antioxidant factors and injure tissues and cells including pancreatic β‐cells, resulting in β‐cell dysfunction which could lead to diabetes [17, 18]; however, studies investigating these associations with SCT are very limited in SSA. This is unfortunate given the very high prevalence of SCT and the rising burden of diabetes in SSA [19].

Although high intensity and infrequent pattern of physical activity have been reported to increase the levels of ROS [20, 21], habitual and moderate physical activity may still improve antioxidant defenses against the ROS in people with SCT [22, 23, 24]. Moderate exercise and an active lifestyle are also useful in primary and secondary prevention of diabetes [25]. However, among SCT carriers, physical exercise may trigger uncomfortable painful experiences in certain environmental conditions such as high temperature or high altitude (due to sickling of red blood cells) [26] leading to reduced physical activity. Reduced physical activity could be associated with high levels of ROS further exacerbating the risk of β‐cell dysfunction among SCT carriers. Since SCD has been a priority, the level of physical activity has been documented in SCD whereas activity in people with SCT appears not to have been reported [27, 28].

HIV infection, with or without the use of antiretroviral therapy (ART), has been reported to increase the risk for β‐cell dysfunction and diabetes since (i) HIV infection induces persistent systemic inflammation, a key role for diabetes pathogenesis [29, 30], and (ii) the use of some ART regimens are reported to be associated with increased oxidative stress and dyslipidemia that could lead to reduced vascular and β‐cell function [29, 31, 32]. In addition, studies have found the level of physical activity to be lower in people living with HIV infection (PLWH) [33]. There are no studies reporting effects of SCT in HIV patients on the level of physical activity, insulin secretion, insulin resistance, and risk of diabetes.

The aim of this study was to investigate SCT associations with physical activity, markers of insulin secretion (β‐cell function), insulin resistance, and glucose tolerance among PLWH and HIV‐uninfected adults.

## METHODS

### Study design and setting

**Fig. 1:**
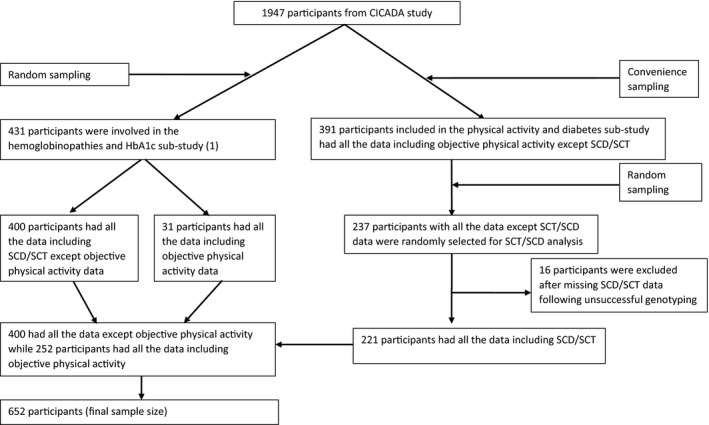
Participant enrolment and inclusion in analysis.

This was a one year cross‐sectional study within the Chronic Infections, Comorbidities and Diabetes in Africa (CICADA) study; a cohort study investigating the burden of and risk factors for diabetes among PLWH and HIV‐uninfected adults in Mwanza, Tanzania [34]. CICADA study recruited 1947 participants from 2016 to 2017. We previously investigated the influence of SCT and other hemoglobinopathies on the levels of hemoglobin A1c (HbA1c) and plasma glucose during an oral glucose tolerance test (OGTT) among 431 participants who were randomly selected from CICADA study [1] and the role of objectively measured physical activity on diabetes among 391 participants, conveniently sampled from CICADA study, in another sub‐study [35]. The current study used data from all 431 participants from the SCT and other hemoglobinopathies sub‐study for SCT/SCD data and, in addition, 237 archived frozen blood samples of participants from the physical activity and diabetes sub‐study were assessed for SCT/SCD (Fig. 1). Subjective physical activity data were retrieved from the main CICADA study and objective physical activity data from the physical activity and diabetes sub‐study.

### Sample size calculation

Based on studies suggesting that reducing oxidative stress could halve diabetes risk [36, 37], we anticipated that SCT would double the risk of having isolated β‐cell dysfunction [12, 13, 14, 15]. Our previous study reported that 37% of people have normal β‐cell function, 27% isolated insulin resistance, 25% isolated β‐cell dysfunction, and 10% have insulin resistance and β‐cell dysfunction [32]. With a conservative assumption that the prevalence of isolated β‐cell dysfunction will increase in people with SCT up to 40%, we would need at least 388 participants without SCT and 97 participants with SCT to demonstrate the anticipated association with 80% power at 5% significance level [38]. To compensate for samples which by coincident would have missed concurrent fasting, 30‐min and 2‐h OGTT or SCT/SCD results and to ensure we would have enough individuals with SCT, we inflated our sample size and recruited 668 participants in total.

### Data collection

#### Demography and socioeconomic data

Data on demography, alcohol intake, and smoking were collected based on WHO STEPS manual questionnaire [39]. Social economic status was divided in tertiles (i.e., lower, middle, and upper). Smoking history was classified as never, past, and current smoking while alcohol intake was classified as never or ever.

#### Anthropometry and body composition

Weight was measured to the nearest 0.1 kg using a digital scale (Seca, Germany), and height was measured to the nearest 0.1 cm using a stadiometer fixed to the wall (Seca, Germany). Body mass index (BMI) was calculated as weight (kg)/(height (m))^2^ and classified as underweight/normal (<25.0 kg/m^2^) or overweight/obese (≥ 25.0 kg/m^2^) [40]. Participants underwent bioelectrical impedance analysis (BIA) to estimate fat mass (kg) and fat‐free mass (kg) using a body composition analyser (Tanita BC418, Tokyo, Japan). These body composition parameters were converted to fat mass index (FMI) and fat‐free mass index (FFMI) by dividing their values by height (m)^2^.

#### HIV status and markers of systemic inflammation

Participants with unknown HIV status were tested according to the national testing algorithm after counseling and consent. HIV testing was done using two rapid antibody tests (SD HIV‐1/2 3.0 SD standard diagnostics Inc, and The Uni‐Gold, Trinity Biotech, IDA Business Park, Bray, Co. Wicklow, Ireland). HIV status was defined as HIV‐negative, HIV‐infected not on ART and HIV‐infected on ART. Additionally alpha‐1‐acid glycoprotein (AGP) and C‐reactive proteins (CRP) were assessed as markers of systemic inflammation using an in‐house sandwich ELISA at Dr. Juergen G. Erhardt laboratory in Germany [41].

#### SCT status

DNA was extracted from whole blood using the QIAMP DNA Mini Kit (Qiagen, Hilden, Germany) according to manufacturer’s instruction. Hemoglobin subtyping for SCT/SCD was performed by polymerase chain reaction –restriction fragment length polymorphism (PCR‐RFLP) and gel electrophoresis as described by Modiano [42].

#### Fasting blood glucose and oral glucose tolerance test

Participants who had fasted for a minimum of 8 h were invited for fasting blood glucose (FBG) test (Hemocue AB, Angelholm, Sweden) and subsequent oral glucose tolerance test (OGTT); tested for glucose level after 30 and 120 min.

#### β‐cell function and insulin resistance

Venous blood samples drawn at the same time as those for glucose assessment (fasting, 30 min and 120 min) were separated and serum stored at −80°C until analysis. ELISA technique was used to assess insulin using dual‐monoclonal antibodies (ALPCO, Salem, NH, USA) in Denmark.

Insulinogenic index (IGI) and homeostatic model assessment for insulin resistance (HOMA‐IR) were selected as markers of β‐cell function and insulin resistance, respectively, based on our previous work [32]. Using cut‐points optimally predicting diabetes reported in our previous paper [32], we derived an overall marker of β‐cell function and insulin resistance by dividing participants into four groups, that is, normal β‐cell function and insulin sensitivity (IGI ≥ 0.71 and HOMA‐IR ≤ 1.9), isolated β‐cell dysfunction (IGI < 0.71 only), isolated insulin resistance (HOMA‐IR >1.9 only), and β‐cell dysfunction and insulin resistance (IGI < 0.71 and HOMA‐IR > 1.9).

#### Physical activity measurements

We collected subjective and objective physical activity data from CICADA and the physical activity and diabetes sub‐study, respectively. Subjective (self‐reported) physical activity data were based on the global physical activity questionnaire (GPAQ) and computed as metabolic equivalent of task minutes per week (MET/week) [43]. Objective physical activity data were collected by a combined heart rate and acceleration monitor (Actiheart, Camtech, Cambridge UK), which provided estimates of physical activity energy expenditure (PAEE) (kj/kg/day), sleeping heart rate (SHR) (beats/min), and average acceleration trunk longitudinal axis (ACCx) (m/s^2^).

#### Ethical considerations

Ethical approval and permission to use the stored blood samples from CICADA study for SCT/SCD analysis was received from the Lake Zone Institution Review Board (LZIRB) (ref no: MR/53/100/624) housed at the National Institute for Medical Research (NIMR), Mwanza, Tanzania.

Since we used stored blood samples informed written or oral consent was not provided.

#### Data management and analyses

Data were captured electronically using CSPro and analyzed in STATA version 13 (Station College, Texas, USA). Participants’ characteristics were presented as mean (SD) or number (percentage) as appropriate. Differences between continuous variables were compared using t‐test and one‐way ANOVA, whereas Chi‐square test was used to compare categorical variables.

β‐cell function and insulin resistance and physical activity levels were the primary outcomes whereas fasting, 30 and 120 min glucose level during an OGTT were secondary outcomes.

We investigated the association of SCT with insulin production and resistance using multinomial logistic regression while linear regression was used to examine the associations of SCT with PAEE, SHR, ACCx, and MET/week based on the self‐reported level of physical activity.

The association of SCT with fasting, 30, and 120 min glucose was investigated using generalized estimating equations (GEE) with Gaussian distribution, identity link (to account for within‐persons correlations of these markers at the 3 time points), and robust standard errors. Scatter plots were used to present the results.

We conducted univariable analyses and variables associated with the outcomes with p < 0.2 were included in multivariable models. Models were first adjusted for age and sex and then further adjusted for other variables.

Multinomial logistic regressions were presented as relative risk ratio (RRR), linear regressions were presented as β‐coefficients, whereas GEE results were presented as marginal means. We presented estimates with 95% CI and p < 0.05 was considered significant. Effect modification by HIV treatment status was explored by fitting interaction terms between ART status and SCT status. Results were stratified by HIV treatment status when test for interactions were significant (p < 0.05).

## RESULTS

### Participants’ selection and characteristics

To comply with the estimated sample size, we tested for SCT/SCD in 668 participants but excluded 16 (2.4%) participants with missing data for SCT/SCD, leaving 652 (97.6%) participants for the analyses (Fig. 1). Compared to participants included for this study, the rest of CICADA population, 1295 participants, had lower average BMI (21.7 vs. 22.3 kg/m^2^, p = 0.003) and FMI (13.13 vs. 14.49 kg/m^2^, p = 0.003) and higher level of CRP (14.5 vs. 9.0 mg/L, p = 0.001) and AGP (1.13 vs. 1.04 g/L, p = 0.04) (Table S1).

The mean age of this study population was 40.0 (SD 11.5) years, 402 (61.7%) were females and 422 (64.7%) were PLWH. Among the PLWH, 80 (18.9%) were already on ART for a median duration of 53 months [34]. SCT (HbAS) was found in 157 (24.1%) participants; no participants had SCD (Table 1).

**Table 1:** Background characteristics of the study population by HIV status

Characteristic	N	HIV‐uninfected (n = 230) Mean (SD) or n (%)	HIV‐infected not on ART (n = 342) Mean (SD) or n (%)	HIV‐infected on ART (n = 80) Mean (SD) or n (%)	p
Age (years)	652	42.6 (12.4)	37.8 (10.5)	44.9 (9.8)	0.01
18–30		39 (16.9)	96 (28.1)	5 (6.3)	<0.001
31–40		71 (30.9)	134 (39.2)	27 (33.8)	
41–50		66 (28.7)	65 (19.0)	29 (36.3)	
>50		54 (23.5)	47 (13.7)	19 (23.6)	
Sex, females	652	132 (57.4)	222 (64.9)	48 (60.0)	0.18
Socio economic tertiles^1^	649				
Lower		52 (22.6)	100 (29.4)	38 (48.1)	<0.001
Middle		74 (32.2)	116 (34.1)	22 (27.9)	
Upper		104 (45.2)	124 (36.5)	19 (24.1)	
Body mass index (kg/m^2^)	652	23.9(4.9)	21.5 (4.2)	20.9 (3.7)	0.01
Normal/underweight		142 (61.7)	288 (84.2)	70 (87.5)	<0.001
Overweight/obesity		88 (38.3)	54 (15.8)	10 (12.5)	
Sickle cell trait status	652				
With SCT		45 (19.6)	83 (24.3)	29 (36.2)	
Without SCT		185 (80.4)	259 (75.7)	51 (63.8)	0.01
Blood glucose level					
Fasting glucose	652	6.6 (1.4)	6.5 (0.8)	7.1 (1.6)	<0.001
30 min glucose^1^	651	8.5 (1.8)	8.4 (1.2)	8.7 (2.5)	<0.001
2 h glucose^1^	651	7.8 (2.7)	8.2 (1.6)	8.3 (2.9)	<0.001
Insulin function status^1^	632				
Normal β‐cell function and insulin sensitivity		86 (38.7)	129 (38.8)	16 (20.8)	<0.001
Isolated β‐cell dysfunction		34 (15.3)	86 (25.8)	21 (27.4)	
Isolated Insulin resistance		74 (33.4)	99 (29.7)	28 (36.4)	
β‐cell dysfunction and insulin resistance		28 (12.6)	19 (5.7)	12 (15.6)	
Physical activity and capacity measures					
Physical activity energy expenditure (kJ/kg/day)^1,^, ^2^	252	48.3 (16.4)	41.8 (20.9)		0.01
Sleeping heart rate (beats/min)^1,^, ^2^	252	60.1 (7.3)	67.1 (10.8)		0.001
Average acceleration, trunk longitudinal axis (m/s^2^)^1,^, ^2^	252	0.1 (0.06)	0.1 (0.1)		0.08
Reported energy expenditure (MET/week)^1^	650	9221 (7142)	9437 (7225)	9227 (7997)	0.43

ART, antiretroviral therapy; MET, Metabolic equivalents.

^1^ Data do not sum to 652 due to missing values.

^2^ HIV infected on ART participants had no data collected for these objective physical activity variables.

### Association of SCT with insulin function status

In unadjusted multinomial regression, we found that people with SCT had a higher risk of isolated β‐cell dysfunction compared to those without SCT (RRR = 1.82, 95% CI: 1.10, 3.01, p = 0.02). The effect estimate did not change with adjustment. There was no association between SCT and isolated insulin resistance or combined β‐cell dysfunction and insulin resistance (Table 2). There was no evidence for effect modification between SCT and HIV treatment status (p = 0.56) with respect to β‐cell function and insulin sensitivity status.

**Table 2:** Multinomial regression of association of sickle cell trait status with insulin function status

Insulin function status	Prevalence, n (%)		Unadjusted analysis		Adjusted analysis^1^	
	No SCT (n = 479)	SCT (n = 153)	RRR (95% CI)	p‐value^2^	RRR (95% CI)	p‐value^3^
Optimal β‐cell function and insulin sensitivity	177 (37.0)	54 (35.3)	Reference		Reference	
Isolated β‐cell dysfunction	91 (19.0)	50 (32.7)	1.82 (1.11, 2.93)	0.01	1.82 (1.10, 3.01)	0.02
Isolated insulin resistance	165 (34.5)	36 (23.5)	0.72 (0.41, 1.13)	0.22	0.72 (0.41, 1.22)	0.21
Combined β‐cell dysfunction and insulin resistance	46 (9.6)	13 (8.5)	0.91 (0.52, 1.83)	0.81	1.04 (0.51, 2.12)	0.91

RRR, Relative Risk Ratio for individuals with sickle cell trait compared to those without SCT.

^1^ Adjusted for age and sex first, then adjusted again for sex, age, body mass index, HIV status, C‐reactive protein, α_1_ acid glycoprotein, smoking and alcohol use status.

^2^ Overall p‐value = 0.01.

^3^ Overall p‐value = 0.01.

### Association of SCT and physical activity

There was no clear directionality in association between SCT and physical activity. Compared to those without SCT and without HIV infection, *ACCx*, an objective marker for physical activity, was significantly lower in people with SCT without HIV infection (β‐coefficient = −0.04 m/s^2^; 95% CI: −0.08, −0.01; p = 0.03) while reported physical activity, a subjective marker for physical activity, was higher (β‐coefficient = 2596 MET/week; 95% CI: 316, 4877; *P* = 0.03). PAEE and SHR had no association with SCT regardless of HIV status. PLWH on ART had no objective physical activity data and showed no association with reported physical activity. There was interaction between SCT and HIV treatment status with regard to markers of physical activity (ACCx and reported physical activity, p = 0.01 and 0.04, respectively), hence results were stratified by HIV status (Table 3).

**Table 3:** Multiple linear regression investigating association between sickle cell trait and physical activity by HIV status and treatment

	Mean (SD)		Unadjusted analysis		Adjusted analysis^1^	
	No SCT	SCT	β‐coefficient (95% CI)	P	β‐coefficient (95% CI)	P
HIV‐negative (n = 230)						
Physical activity energy expenditure (kJ/kg/day)	41.1 (18.3)	41.4 (16.2)	2.3 (−13.9, 9.2)	0.69	–1.4 (−13.2, 10.5)	0.82
Sleeping heart rate (beats/min)	60.3 (7.0)	59.0 (9.4)	−1.3 (−6.4, 3.9)	0.63	−0.3 (−4.5, 5.1)	0.89
Average acceleration, trunk longitudinal axis (m/s^2^)	0.13 (0.06)	0.09 (0.04)	−0.03 (−0.07, 0.01)	0.11	− 0.04 (−0.08, −0.01)	0.03
Reported energy expenditure (MET/week)	8703 (6587)	11336 (8841)	2632 (312, 4952)	0.03	2596 (316, 4877)	0.03
HIV‐infected not on ART (n = 342)						
Physical activity energy expenditure (kJ/kg/day)	34.0 (18.8)	40.5 (18.4)	4.5 (−3, 12)	0.24	5.7 (−1.6, 13)	0.12
Sleeping heart rate (beats/min)	67.2 (10.9)	66.6 (10.6)	−0.7 (−4.6, 3.2)	0.72	0.6 (−2.8, 4)	0.73
Average acceleration, trunk longitudinal axis (m/s^2^)	0.09 (0.07)	0.11 (0.05)	0.02(−0.01, 0.04)	0.19	0.01 (−0.01, 0.04)	0.18
Reported energy expenditure (MET/week)	9533 (7342)	9140 (6883)	−392 (−2188,1402)	0.67	−642 (−2462, 1178)	0.49
HIV‐infected on ART (n = 80)^2^						
Physical activity energy expenditure (PAEE), (kJ/kg/day)^2^	–		–		–	
Sleeping heart rate (SHR), (beats/min)^2^	–		–		–	
Average acceleration, trunk longitudinal axis (ACCx), (m/s^2^)^2^	–		–		–	
Reported energy expenditure (MET/week)	9123 (7724)	9403 (8593)	285 (−3440, 4011)	0.88	123 (−3583, 3830)	0.95

^1^ Adjusted for age and sex then adjusted again for sex, age, body mass index, C‐reactive protein and α_1_ acid glycoprotein.

^2^ HIV infected on ART participants had no data collected for PAEE, SHR and ACCx.

### Association of SCT with blood glucose

**Fig. 2:**
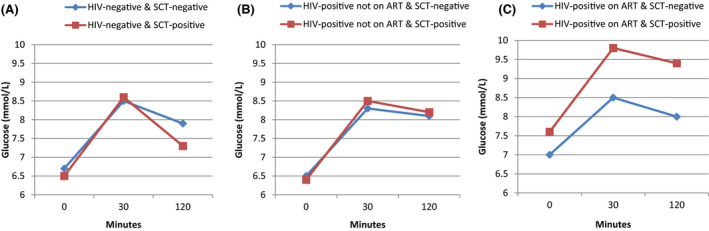
(A) Marginal means of oral glucose tolerance test (OGTT) according to sickle cell trait (SCT) status among HIV‐negative participants (230 participants). Using generalized estimated equation (GEE), we found no difference in the distribution of OGTT between people with SCT (185 participants) and those without SCT (45 participants) at 0 (6.5 mmol/L 95% CI: 5.9, 7.1 vs. 6.7 mmol/L; 95% CI: 6.3, 7.1; p = 0.51), 30 (8.6 mmol/L; 95% CI: 7.9, 9.2 vs. 8.5 mmol/L; 95% CI: 8.0, 8.9; p = 0.75) and 120 min (7.3 mmol; 95% CI: 6.8, 7.8 vs. 7.9 mmol/L; 95% CI: 7.3, 8.5; p = 0.09). (B) Marginal means of OGTT according to SCT status among people living HIV infection (PLWH) not on antiretroviral therapy (ART) (342 participants). Using GEE, we found no difference in the distribution of OGTT between people with SCT (83 participant) and those without SCT (259 participants) at 0 (6.4 mmol/L; 95% CI: 6.2, 6.6 vs. 6.4 mmol/L 95% CI: 6.2, 6.5; p = 0.93), 30 (8.5 mmol/L; 95% CI: 8.1, 8.8 vs. 8.2 95% CI: 8.0, 8.5; p = 0.21) and 120 min (8.1 mmol/L; 95% CI: 7.7, 8.5 vs. 8.0 mmol/L; 95% CI: 7.8, 8.3; p = 0.93). (C) Marginal means of OGTT according to SCT status among PLWH on ART (80 participants). By GEE, we found a significant difference in the distribution of OGTT between people with SCT (29 participants) and those without SCT (51 participants) at 30 min (9.7 mmol/L; 95% CI: 8.3, 11.2 vs. 8.5 mmol/L; 7.7, 9.2; p = 0.03) while OGTT at 0 and 120 min were marginally higher (7.6 mmol/L; 95% CI: 6.6, 8.6 vs. 6.9 mmol/L 95% CI: 6.5, 7.5; p = 0.07 and 9.3 mmol/L; 95% CI: 7.4, 11.2 vs. 7.9 mmol/L; 95% CI: 7.3, 8.6; p = 0.08 respectively).

Unadjusted GEE analysis revealed that 30‐minutes glucose level during OGTT was higher in people with SCT compared to those without SCT on ART (9.7 mmol/L; 95% CI: 8.3, 11.2 vs. 8.5 mmol/L; 95% CI: 7.7, 9.2; p = 0.03). In adjusted analysis, 30 min blood glucose during the OGTT remained higher in people with SCT compared to those without SCT on ART (overall p = 0.04) (Fig. 2C). In addition, in adjusted analysis, people with SCT had marginally higher fasting and 120 min glucose levels compared to those without SCT on ART individuals (7.6 mmol/L; 95% CI: 6.6, 8.6 vs. 6.9 mmol/L; 95% CI: 6.5, 7.5; p = 0.07 and 9.3 mmol/L; 95% CI: 7.4, 11.2 vs. 7.9 mmol/L; 95% CI: 7.3, 8.6; p = 0.08, respectively) (Fig. 2C). No association was observed between SCT and fasting, 30 and 120 min glucose in either HIV‐uninfected or HIV‐infected not on ART individuals (Fig. 2A,B). There was interaction between HIV treatment status and SCT with regard to glucose level (at fasting, 30 and 120 min, *P* = 0.04, 0.03 and 0.03, respectively), hence results were stratified by HIV treatment status.

## DISCUSSION

We hypothesized that people with SCT are at higher risk of diabetes due to β‐cells dysfunction caused by oxidative stress exacerbated by lower level of physical activity. We did find a higher risk of β‐cell dysfunction in individuals with SCT irrespective of their HIV status, but the data did not support a strong impact of SCT on their habitual physical activity level. We also observed higher levels of blood glucose levels among individuals with SCT on ART, suggesting an increased risk of future diabetes.

Regardless of HIV status, individuals with SCT had higher risk of β‐cell dysfunction which has also been seen among individuals with SCD [44], but we have not identified previous studies relating SCT with β‐cell dysfunction or insulin resistance. Oxidative stress could mediate the risk for β‐cell dysfunction among individuals with SCT [17], but we did not assess oxidative stress as it was beyond the scope of this study.

Regular physical activity has been reported to strengthen antioxidant defense systems [23]; hence, elevated level of oxidative stress may not be regulated well if the level of physical activity is low. To understand the relationship between SCT and the level of physical activity, we used both objective and subjective physical activity measurements. We found no obvious association between SCT and physical activity measurements. Objective physical activity data indicated a lower level of ACCx in people with SCT without HIV infection, while PAEE and SHR showed no association with SCT regardless of HIV status. Unexpectedly, self‐reported physical activity measurement was higher in people with SCT without HIV infection.

We included PLWH not on ART and PLWH on ART to assess the interaction effect of HIV infection or ART with SCT on β‐cell function and diabetes outcome. Individuals with SCT on ART had significantly higher level of 30 min glucose compared to individuals without SCT on ART. Fasting and 120 min glucose level were also marginally higher, though not significant, in individuals with SCT on ART compared to individuals without SCT on ART. These findings suggest that coexistence of SCT, HIV infection, and ART use may increase risk for diabetes through a synergistic effect or additive effects in increasing plasma glucose level, because (i) SCT individuals have overproduction of ROS, the common cause of pancreatic β‐cell damage [17] and (ii) PLWH on ART have been reported to have many risk factors for diabetes development, that is, oxidative stress, obesity and lipidemia [45, 46, 47, 48]. The strongest effect on blood glucose was seen at 30 min after OGTT, indicating an impaired early insulin secretion, that is, β‐cell dysfunction. Although the etiology for diabetes could not be established in this cross‐sectional study, long‐term oxidative stress from SCT is a likely stress on the β‐cells and should be explored in more mechanistic studies, as this may trigger a non‐classical diabetes from the combination of relative insulin insufficiency and insulin resistance from obesity, inactivity, and dyslipidemia.

Despite its known complications that are thought to have effect on glucose level, data reporting the role of SCT alone or SCT with co‐morbidities, for example, HIV infection on glucose level are very limited. We found one study that reported SCT among HIV‐uninfected population to have no relation with diabetes [49], similar to what we have reported on the association of SCT on glucose among HIV‐uninfected individuals. Taken together, these findings suggest SCT could induce β‐cell dysfunction without causing major impact on glucose level unless augmented by other medical conditions that also contribute to diabetes development such as HIV infection and ART use. Previously SCT has been reported only to worsen diabetes complications (e.g., retinopathy, proteinuria, vascular disease, etc.) [50] rather than increasing risk of developing diabetes. However, in the current analysis, we have reported that SCT could also increase the risk of having diabetes especially when it coexists with other medical conditions that also have impacts on glucose level. SCT affects more than 300 million people worldwide [51], but since it present with no symptoms, SCT has been among the neglected blood disorder compared with SCD. Our study provides data on the potential adverse effects of SCT on β‐cell function arguing for the need of more research on the role of SCT on metabolic disease development.

This study has a number of strengths. First, we attained a high prevalence of SCT within a modest sample size because our study was nested in a well‐characterized cohort from north‐western region of Tanzania, a geographical area reported to have high prevalence of SCD/ SCT. Second, in addition to reported physical activity data, we used objective physical activity data which are more reliable and less biased than self‐reported activity. Third, we assessed blood glucose level at different time points (i.e., fasting, 30 and 120 min). The inclusion of 30 min blood glucose level (a good predictor for diabetes occurrence [52]) to determine early insulin secretion helped to understand the combined effect of SCT and ART use on glucose, an observation we would have missed if we had only used fasting and 120 min.

This study was limited by being cross‐sectional; therefore, we were unable to confirm causal associations between SCT and study outcomes. Second, to save time and be cost‐efficient, participants for this study were not selected randomly from the community, but based on existing material collected as part of the CICADA study, which also had a higher proportion of HIV infection compared with the background population limiting generalizability to the general population. Third, although physical activity data were important for this study, we only assessed activity over a few days, while development of β‐cell dysfunction must have occurred over many years. Fourth, PLWH on ART were only 80 and a larger sample size of this population might have turned the marginal association of SCT with fasting or 120 min OGTT into a significant association. Lastly, objective physical activity data for PLWH on ART were not collected; hence, we lacked data on the association between objectively collected physical activity and people with SCT on ART.

## CONCLUSION

The risk of β‐cell dysfunction seems to be higher among people with SCT. However, β‐cells dysfunction in SCT individuals may not proceed to overt diabetes unless accompanied by a second hit such as a medical condition separately increasing the risk for diabetes, for example, HIV infection, ART usage and insulin resistance from obesity, and sedentary lifestyle. We suggest further studies to investigate and quantify biomarkers of oxidative stress in people with SCT on ART, as this was missing in our data.

Future longitudinal studies should help identify the actual cause and mechanism behind diabetes development among SCT on ART individuals. Cost‐effective screening programs for SCT and diabetes in PLWH on ART should be considered in regions with high prevalence of SCT as it may help preventing diabetes from lifestyle changes and also impact diabetes management which may be different for people with SCT.

## CONFLICT OF INTERESTS

All authors declare that they have no competing interests.

## FUNDING

This work was supported through the DELTAS Africa Initiative grant # DEL‐15‐011 to THRiVE‐2 and the Ministry of Foreign Affairs of Denmark through a grant administered by the Danida Fellowship Centre (grant: 16‐P01‐TAN). The DELTAS Africa Initiative is an independent funding scheme of the African Academy of Sciences (AAS)’s Alliance for Accelerating Excellence in Science in Africa (AESA) and supported by the New Partnership for Africa’s Development Planning and Coordinating Agency (NEPAD Agency) with funding from the Wellcome Trust grant #107742/Z/15/Z and the UK government. The views expressed in this publication are those of the author(s) and not necessarily those of AAS, NEPAD Agency, Wellcome Trust, UK government or Danida Fellowship Centre.

## Supporting information


**Table S1**. Comparison of full cohort characteristic with sub‐study.
