# Supplementary material for: Association of sickle cell trait with β‐cell dysfunction and physical activity in adults living with and without HIV in Tanzania
Source: APMIS. 2022 Mar 1;130(4):230–9. doi: 10.1111/apm.13214 (PMC9314065; doi:10.1111/apm.13214)
Supplement: Supplementary file 1 — Table S1. Comparison of full cohort characteristic with sub‐study. [file APM-130-230-s001.pdf]

Supplementary Table 1: Comparison of full cohort characteristic with sub-study[1,2]

Variables	Overall (N=1947)	In sub-study (N=652), Mean (SD) or n (%)	Not in sub-study (N=1295), Mean (SD), n (%)	P
**Age (yrs)**	1947	40.4 (11.5)	40.9 (12.1)	0.39
18-30		140 (21.5)	280 (21.6)	0.09
31-40		232 (35.6)	391 (30.2)	
41-50		160 (24.5)	361 (27.9)	
>50		120 (18.4)	263 (20.3)	
**Sex, female**	1947	402 (61.7)	755 (58.3)	0.16
**Smoking**	1942			
Never		500 (76.9)	974 (75.4)	0.76
Past		92 (14.2)	194 (15.0)	
Current		58 (8.9)	124 (9.6)	
**Alcohol**	1942			
Never		174 (26.8)	379 (29.3)	0.24
Ever		476 (73.2)	913 (70.7)	
**Body mass index (kg/m^2)**	1946	22.3 (4.5)	21.7 (4.5)	0.003
Normal		500 (76.7)	1056 (81.6)	0.01
Overweight/obesity		156 (23.3)	238 (18.4)	
**Fat mass index (kg/m^2)**	1900	14.49 (9.6)	13.13 (9.2)	0.003
**Fat-free mass index (kg/m^2)**	1900	44.6 (7.4)	44.6 (7.6)	0.92
**C-reactive protein (mg/L)**	1919	9.0 (23.1)	14.5 (37.1)	0.001
**α_1-acid glycoprotein (g/L)**	1919	1.04 (0.77)	1.13 (0.94)	0.04

[1]Data are number (%) unless specifically indicated as mean (SD)
[2]Data do not sum to 1947 due to missing values
